# A randomised trial of second-line hormone vs single agent chemotherapy in tamoxifen resistant advanced breast cancer.

**DOI:** 10.1038/bjc.1992.277

**Published:** 1992-08

**Authors:** A. R. Dixon, L. Jackson, S. Chan, J. Haybittle, R. W. Blamey

**Affiliations:** City Hospital, Nottingham, UK.

## Abstract

Sixty patients with advanced breast cancer unresponsive to tamoxifen have been randomised to receive four course of mitozantrone, 14 mg m-2 (n = 30) intravenously every 3 weeks (9 weeks total) or megesterol acetate, 160 mg bd (n = 30). One in three patients (11 from each group) had substantial disease control for a minimum period of 6 months i.e., lack of progression; seven patients (23%) showed objective response to mitozantrone compared to four (13%) receiving megesterol. Non-progressive disease occurred in all sites, including visceral metastases and receptor negative patients. There were no significant differences between treatment groups in the median time (5 months each) to disease progression response duration or survival (13 months megesterol, 11 months mitozantrone) from commencing second-line therapy. Toxicity was considerably higher in the mitozantrone group. Second-line hormonal therapies can produce similar therapeutic results as those achieved from a short course of a 'short option' single agent cytotoxic in patients who were previously thought hormone insensitive. Provided that the patient does not have life threatening disease a trial of megesterol acetate is worth consideration in that it does not prejudice subsequent response to combination cytotoxic chemotherapy.


Br. J. Cancer (1992), 66, 402-404 © Macmillan Press Ltd., 1992

### A randomised trial of second-line hormone *vs* single agent chemotherapy in tamoxifen resistant advanced breast cancer

A.R. Dixon, L. Jackson, S. Chan, J. Haybittle & R.W. Blamey

*City Hospital, Nottingham NG5 1PB, UK.*

**Summary** Sixty patients with advanced breast cancer unresponsive to tamoxifen have been randomised to receive four course of mitozantrone, 14 mg $m^{-2}$ ($n = 30$) intravenously every 3 weeks (9 weeks total) or megesterol acetate, 160 mg bd ($n = 30$). One in three patients (11 from each group) had substantial disease control for a minimum period of 6 months i.e., lack of progression; seven patients (23%) showed objective response to mitozantrone compared to four (13%) receiving megesterol. Non-progressive disease occurred in all sites, including visceral metastases and receptor negative patients. There were no significant differences between treatment groups in the median time (5 months each) to disease progression/response duration or survival (13 months megesterol, 11 months mitozantrone) from commencing second-line therapy. Toxicity was considerably higher in the mitozantrone group.

Second-line hormonal therapies can produce similar therapeutic results as those achieved from a short course of a 'short option' single agent cytotoxic in patients who were previously thought hormone insensitive. Provided that the patient does not have life threatening disease a trial of megesterol acetate is worth consideration in that it does not prejudice subsequent response to combination cytotoxic chemotherapy.

With current therapies available no patients is cured of advanced breast cancer; treatment is palliative being directed towards the relief of symptoms without compromise to the quality of life that remains. Palliation depends upon a balance between objective regression of symptomatic tumour burden and toxicity of treatment. Within most centres of the UK, endocrine manipulation remains the preferred initial treatment provided death is not imminent. Tamoxifen is the most popular initial treatment choice in postmenopausal patients. Those patients that fail to respond to tamoxifen have a very poor prognosis (Williams *et al.*, 1986).

We have compared the synthetic progestogen, megesterol acetate (Megace®, Bristol Myers, UK) against a 'soft option' single-agent cytotoxic, mitozantrone (Novantrone®, Lederle, UK) in terms of 1 year progression free rates and survival in a group of patients who had shown no therapeutic response to tamoxifen. It was not our intention to compare response rates.

### Patients and methods

Sixty postmenopausal advanced breast cancer patients who had relapsed within 6 months of commencing tamoxifen were entered into this prospective study. None had received previous adjuvant therapies. Each was randomised to receive either megesterol acetate, 160 mg bd. or mitozantrone 14 mg $m^{-2}$ every 3 weeks for four courses (i.e., for 9 weeks from day 1). Thirty patients were randomised into each treatment arm. Patients with brain metastases or jaundice were excluded. Pre-treatment performance status had to be two or less on the World Health Organisation scale (World Health Organisation, 1979). Patients failing to respond to either second-line therapy then went on to receive combination CMF if appropriate; patients with non-progressive disease after four cycles of mitozantrone were randomised to stop cytotoxic therapy or change to CMF. Ethical committee approval and informed consent from all patients was obtained.

The median age was 64 years (43–78) for those patients receiving megesterol acetate and 61 years (42–75) for mitozantrone. Major sites of disease are shown in Table I. Chemotherapy continued unless the while cell count was $<2.5 \times 10^9$ l, platelets $<100 \times 10^9 l^{-1}$ or haemoglobin $<10$ g $dl^{-1}$. Response and toxicity were determined using standard UICC (Hayward *et al.*, 1979) and WHO criteria (World Health Organisation, 1979). The British Breast Group's recommendation (British Breast Group, 1974) that the minimum duration of remission be 6 months was also adhered. The patients ECOG/WHO performance status was recorded prior to treatment and at each assessment thereafter.

Primary tumour steroid receptor status was known in 51 patients; the oestrogen receptor assays having been performed at the Tenovus Institute, Cardiff using the commercial ER-enzyme immunoassay (Abbot ER-Eia monoclonal). Oestrogen receptor was considered positive when a value $>15$ fmol mg/cytosol protein was obtained (Walker *et al.*, 1989). The histological grade of the primary tumour (Elston, 1987) was known in 54 patients.

Actuarial survival analysis was performed using the statistical package SPSSX-21 life table analysis (SPSS, 1986) which calculates Gehan's generalised Wilcoxon rank test for censored data (Lee & Desu, 1972). During the course of the study it became evident that there was no early advantage for the group randomised to single agent chemotherapy. Having sought statistical advice (JH) the trial was abandoned once sufficient events had occurred to allow for sufficient statistical power in its analysis. With an expected response rate of 15–17% to megesterol in this treatment setting (Blackledge *et al.*, 1986; Robertson *et al.*, 1989*b*) and 30–35% to mitozantrone (Morridsen *et al.*, 1985; Harris *et al.*, 1990) we calculated that to have a 90% (9 in 10) chance (power) of detecting an improvement from 15% to 35% in progression-free rates at 1 year we would need 49 events in total (Freedman, 1982), the number of events observed, assuming logrank comparison between time to progression curves.

### Results

The overall objective response rate (18%) was poor; four partial responses followed treatment with megesterol and seven to mitozantrone. There were no complete responders in either treatment arm. Stable disease of a minimum duration of 6 months was recorded in seven patients (23%) treated by megesterol acetate and four (13%) treated by mitozantrone; 38 patients (63%) had progressive disease. Thus, one in three patients in each group had substantial disease control i.e., lack of progression for $\geqslant 6$ months. Responses (partial and static) were observed in all disease sites with the response rate in liver secondaries, 3/8 (37%) to megesterol similar to that seen with mitozantrone, 4/9 (44%). The response rates

Correspondence: A.R. Dixon, Professorial Unit of Surgery, City Hospital, Hucknall Road, Nottingham NG5 1PB, UK.
Received 1 February 1992; accepted 9 March 1992.

observed in other sites included: bone 5/12 (41%) for megesterol and 5/14 (36%) for mitozantrone; lung 2/5 (40%) for megesterol and 2/7 (28%) for mitozantrone; and 1/3 stage IIIs treated by megesterol. Responsive disease and disease stabilisations were seen in both receptor positive (10) and negative (7) tumours (Table I).

Pretreatment characteristics (site of metastases, tumour grade and oestrogen receptor status) known to effect prognosis once metastases appear were evenly distributed amongst the two treatment groups (Table II). There were no differences in the median survival from starting second-line treatments or time to disease progression as measured by log-rank analysis (Figures 1 and 2). Confidence limits on the difference in 1 year progression-free rates (diff = 0.2315 − 0.1164 i.e., 0.1151) were: 95% confidence limits −0.095 to 0.325; 90% confidence limits −0.061 to 0.291. The negative lower limits are in favour of megesterol acetate i.e., there is only a 1 in 20 chance of the difference being more than 29% in favour of mitozantrone.

Although quality of life was not formally measured, an improvement in performance status was reported in eight patients receiving chemotherapy and 11 megesterol. Fourteen patients (46%) experienced nausea and vomiting (grade II in seven, grade III in four) to the chemotherapy and three developed stomatitis (grade I). All were prescribed prophylactic metaclopramide (20 mg 8 hourly) as required. Alopecia was reported by 12 (40%) patients, four of whom required a wig. Ten patients (33%) reported no side effects of mitozantrone. Myelosuppression sufficient to cause a delay in the 3-weekly regimen and dose reduction occurred in four patients, three of whom required a blood transfusion. One patient developed an acute anaphylaxis requiring adrenaline, hydrocortisone and $O_2$, 2 h following her fourth dose of mitozantrone. There were no cardiac toxic effects requiring dose modification.

The toxicity and side effects of megesterol acetate were minimal. The most commonly reported side-effects were sweating/hot flushes (four patients), increased appetite and weight gain. Four patients experienced a $<10\%$ weight gain and one a $>10\%$ gain, the latter requiring a dose reduction to 80 mg bd. The increased appetite was particularly welcomed by three patients. There were no cases of hypercalcaemia, venous thrombosis or cardiac failure due to fluid retention.

**Table I** Receptor status and response

| | *Response* | *Megesterol acetate* | *Mitozantrone* |
|---|---|---|---|
| ER +ve | PR | 2 | 2 |
| | SD | 4 | 2 |
| | Prog | 2 | 2 |
| ER −ve | PR | 1 | 3 |
| | SD | 1 | 2 |
| | Prog | 14 | 16 |
| Unknown | PR | 1 | 2 |
| | SD | 2 | – |
| | Prog | 3 | 1 |

**Table II** Pretreatment characteristics

| | *Megesterol acetate* | *Mitozantrone* |
|---|---|---|
| *Site of disease* | | |
| Stage III | 3 | 4 |
| Bone | 13 | 10 |
| Pulmonary | 2 | 3 |
| Bone & Pulmonary | 3 | 4 |
| Visceral | 9 | 9 |
| *Tumour grade* | | |
| Grade 1 | 2 | 3 |
| Grade 2 | 11 | 12 |
| Grade 3 | 12 | 14 |
| *Oestrogen receptor status* | | |
| ER +ve | 8 | 6 |
| ER −ve | 16 | 21 |

**Figure 1** Probability of survival from commencing second-line megesterol or mitozantrone. –□–, Megace; –■–, mitozantrone.

**Figure 2** Duration of response in patients receiving mitozantrone or megesterol. –□–, Megace; –■–, mitozantrone.

### Discussion

The conventional treatment of those patients failing to show a response to first-line tamoxifen is cytotoxic chemotherapy, particularly with visceral disease (Henderson, 1986). Before dismissing the option of further hormonal therapies, consideration has to be given to the fact that long-term remissions and improved survival are rarely seen in advanced breast cancer treated with cytotoxic chemotherapy (Powels *et al.*, 1980), treatment is aimed at achieving palliative remission of symptoms. We have compared for the first time a second-line hormonal therapy against a 'soft option' single agent cytotoxic in apparent hormone resistant breast cancer, irrespective of disease site provided that the disease was not fulminate. Comparison was made between megesterol acetate and mitozantrone, a single agent with substantial activity and low toxicity (Harris *et al.*, 1990).

Phase II studies have reported on mitozantrone as a single agent in advanced breast cancer with widely varying results; a literature review (Leyden *et al.*, 1984) reporting a mean response rate of only 17% (range 4–44%). The larger published series are consistent in reporting objective response rates of approximately 30% (Morrisden *et al.*, 1985; Harris *et al.*, 1990). The response rate in our own series (Robertson *et al.*, 1989) of heavily pretreated patients, measured at 3 and 6 month assessments (14% and 15% respectively) lies at the lower end of the reported range; 22% of patients in our series had static disease.

Megesterol acetate has been used within our unit (Robertson *et al.*, 1989*b*) as a second-line therapy following tamoxifen failure. Of 93 patients that had shown an initial objective response/static disease after 6 months treatment with tamoxifen, upon later relapse, 62% of these subsequently showed non-progression of their disease following 6 months of megesterol. Similar response rates have been observed by other workers (Ross *et al.*, 1982; Blackledge *et al.*, 1986). Only 17% of the 66 patients who had progressed within 6 months of commencing tamoxifen had non-progressive disease when subsequently treated with megesterol (Robertson *et al.*, 1989*b*). As this latter result was similar to our experience with the more toxic mitozantrone, it seemed sensible to compare the two contrasting second-line treatments on a randomised basis.

Although the number of patients entering into this study is small there is clearly no great advantage in terms of progression-free survival to the single-agent cytotoxic, indeed the negative lower confidence limits favours megesterol, the number of events being more important than the actual number of recruited patients. Megesterol acetate appears to be as effective in controlling disease progression as a short 9 week course of single-agent mitozantrone in patients without immediate life threatening disease who had failed to respond to tamoxifen; megesterol gave disease control of more than 6 months duration in one patient in three. Toxicity was substantially lower with megesterol, giving rise to a higher quality of palliative response in those patients who achieved objective regression/stabilisation of their symptomatic disease. Within the setting of an advanced breast cancer clinic the side effects of increased appetite and weight gain associated with megesterol are frequently desirable.

### References

BLACKLEDGE, G.R.P., LATIEF, T., MOULD, J.J., SPOONER, D. & MORRISON, M. (1986). Phase II evaluation of megesterol acetate in previously treated patients with advanced breast cancer: relationship of response to previous treatment. *Eur. J. Cancer Clin. Oncol.*, **22,** 1091.

BRITISH BREAST GROUP (1974). Assessment of response to treatment in advanced breast cancer. *Lancet*, **ii,** 38.

ELSTON, C.W. (1987). Grading of invasive carcinoma of the breast. In *Diagnostic Histopathology of the Breast*. Page, D.L. & Anderson, T.J. (eds), p. 300. Churchill Livingstone: Edinburgh.

FREEDMAN, L.S. (1982). *Stat. Med.*, **1,** 21.

HARRIS, A.L., CANTWELL, B.M.J., CARMICHAEL, J. & 5 others (1990). Comparison of short-term and continuous chemotherapy (mitozantrone) for advanced breast cancer. *Lancet*, **i,** 186.

HAYWARD, J.L., CARBONE, P.P., HEUSON, J.C., KUMAOKA, S., SEGALOFF, A. & RUBENS, R.D. (1977). Assessment of response to therapy in advanced breast cancer. *Cancer*, **39,** 1289.

HENDERSON, I.C. (1986). The treatment of advanced breast cancer. In *Recent Advanced in Clinical Oncology*. Williams, C.J. & Whityehouse, J.M.A. (eds), p. 135. Churchill Livingstone: Edinburgh.

LEE, E.T. & DESU, M.M. (1972). A computer program for comparing two samples with censored data. *Comp. Prog. Biomed.*, **2,** 315.

LEYDEN, M., CHENG, Z., COLLINS, J., RUSSELL, I., ANDREWS, J. & SULLIVAN, J. (1984). Mitozantrone tratment in advanced breast cancer. *Aust. N.Z. J. Surg.*, **54,** 21.

MOURRIDSEN, H.T., CORUBLET, M., STUART-HARRIS, R.C. & 7 others (1985). Mitozantrone as first-line chemotherapy in advanced breast cancer: results of a collaborative European study. *Invest. New Drugs*, **3,** 139.

POWELS, T.J., SMITH, I.E., FORD, H.T., COOMBES, R.C., JONES, J.M. & GAZET, J.-C. (1980). Failure of chemotherapy to prolong survival in a group of patients with metastatic breast cancer. *Lancet*, **i,** 580.

ROBERTSON, J.F.R., WILLIAMS, M.R., TODD, J.H. & BLAMEY, R.W. (1989*a*). Mitozantone – a useful palliative therapy in advanced breast cancer. *Am. J. Clin. Oncol.*, **12,** 393.

ROBERTSON, J.F.R., WILLIAMS, M.R., TODD, J.H., NICHOLSON, R.I., MORGAN, D.A.L. & BLAMEY, R.W. (1989*b*). Factors predicting the response of patients with advanced breast cancer to endocrine (megace) therapy. *Eur. J. Clin. Oncol.*, **25,** 469.

ROSS, M.B., BUZDAR, A.V. & BLUMENSCHENEIN, G.R. (1982). Treatment of advanced breast cancer with megesterol acetate after therapy with tamoxifen. *Cancer*, **49,** 413.

SPSSX (1986). *SPSSx User's Guide*. McGraw-Hill: New York.

WALKER, K.J., BOUZUBAR, N., ROBERTSON, J. & 6 others (1989). Immunocytochemical localisation of estrogen receptor in human breast tissue. *Cancer*, **48,** 6517.

WILLIAMS, M.R., TODD, J.H. & NICHOLSON, R.I. (1986). Survival patterns in hormone treated advanced breast cancer. *Br. J. Surg.*, **73,** 752.

WORLD HEALTH ORGANIZATION (1979). *WHO Handbook for Reporting of Cancer Treatment*. Geneva.


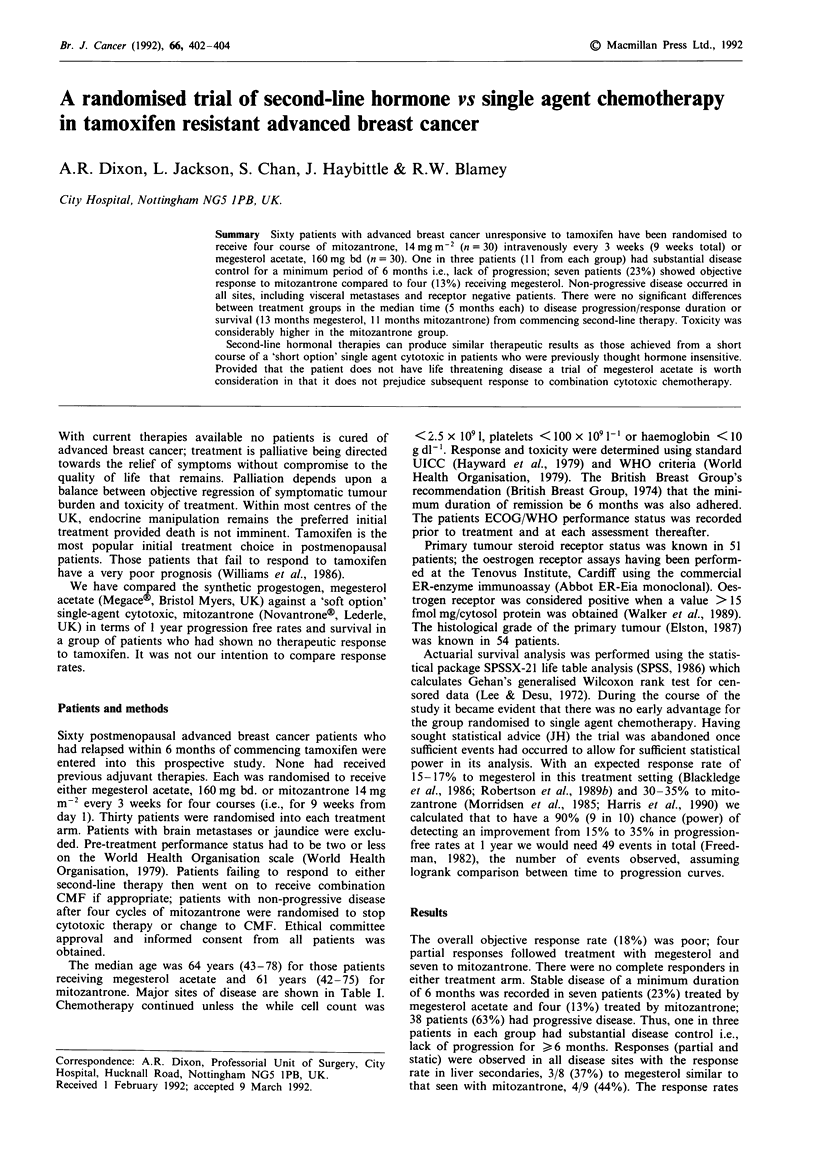

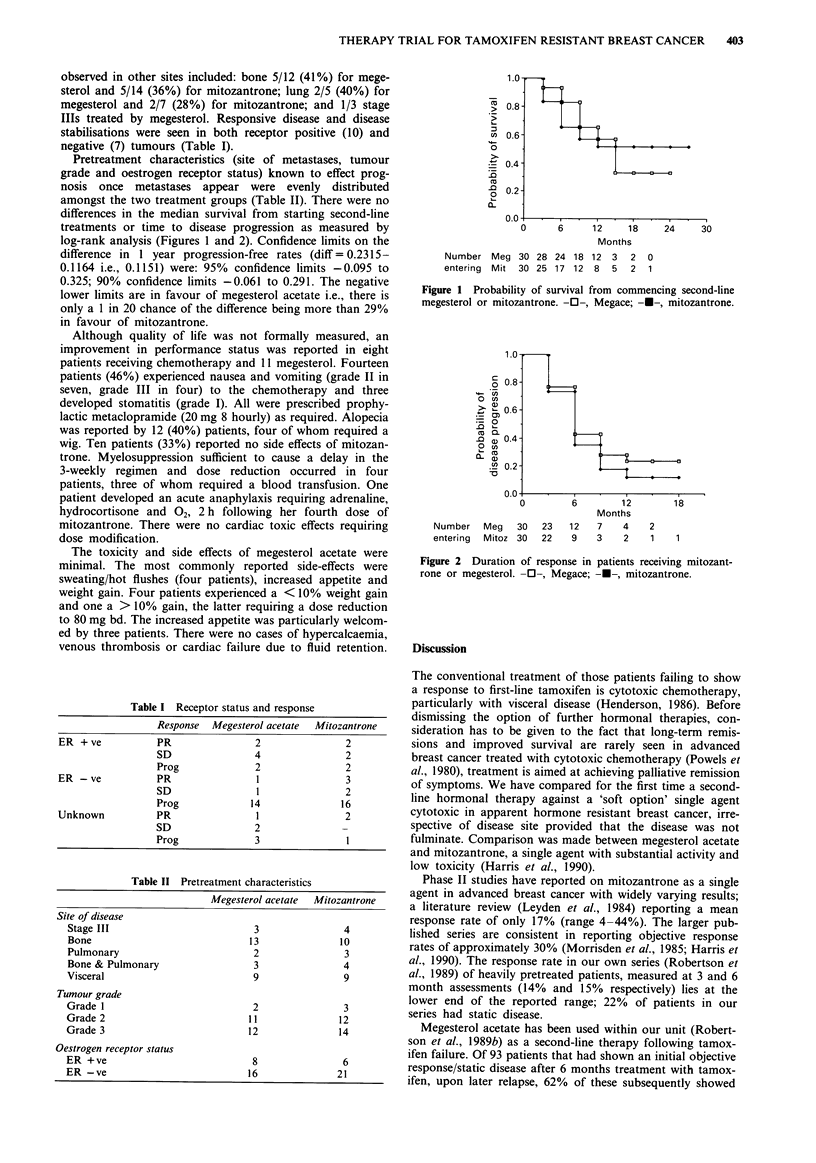

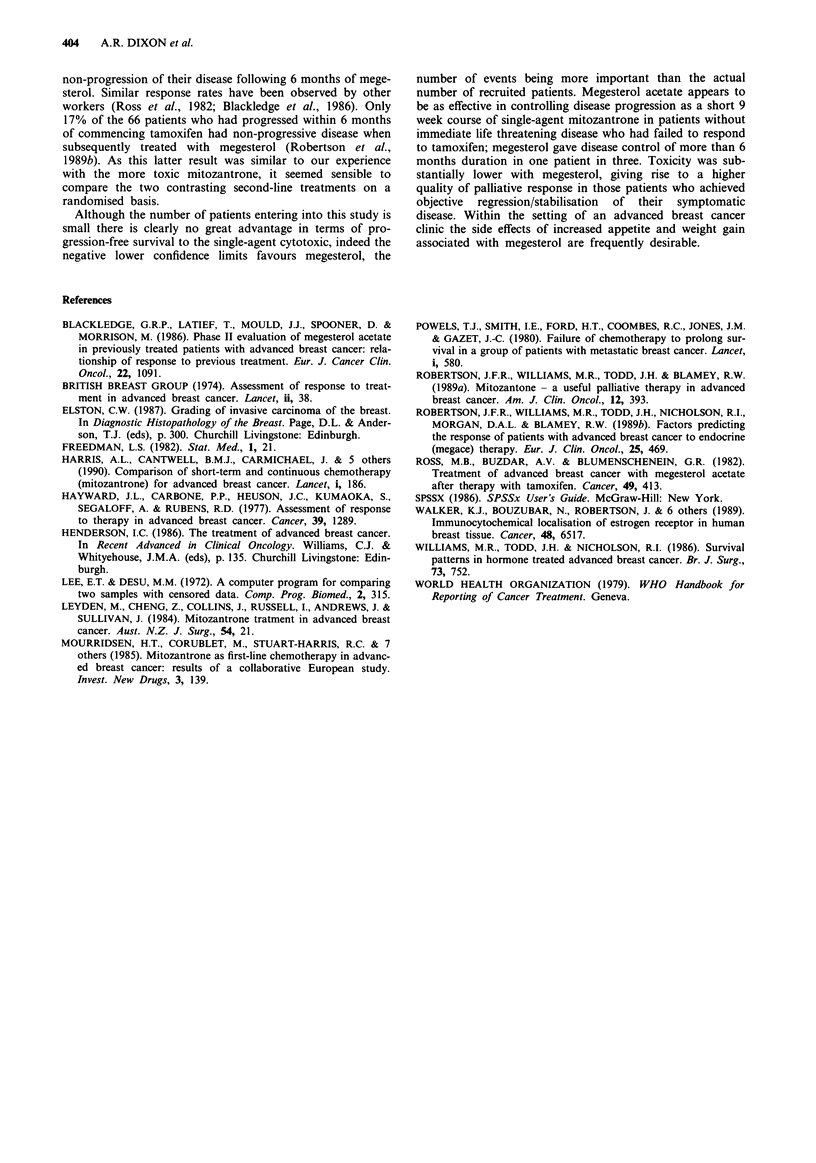


## References

[OCR_00346] Blackledge G. R., Latief T., Mould J. J., Spooner D., Morrison M. (1986). Phase II evaluation of megestrol acetate in previously treated patients with advanced breast cancer: relationship of response to previous treatment.. Eur J Cancer Clin Oncol.

[OCR_00363] Harris A. L., Cantwell B. M., Carmichael J., Wilson R., Farndon J., Dawes P., Ghani S., Evans R. G. (1990). Comparison of short-term and continuous chemotherapy (mitozantrone) for advanced breast cancer.. Lancet.

[OCR_00368] Hayward J. L., Carbone P. P., Heuson J. C., Kumaoka S., Segaloff A., Rubens R. D. (1977). Assessment of response to therapy in advanced breast cancer: a project of the Programme on Clinical Oncology of the International Union Against Cancer, Geneva, Switzerland.. Cancer.

[OCR_00379] Lee E. T., Desu M. M. (1972). A computer program for comparing K samples with right-censored data.. Comput Programs Biomed.

[OCR_00382] Leyden M., Cheng Z. M., Collins J., Russell I., Andrews J., Sullivan J. (1984). Mitoxantrone treatment in advanced breast cancer.. Aust N Z J Surg.

[OCR_00387] Mouridsen H. T., Cornbleet M., Stuart-Harris R., Smith I., Coleman R., Rubens R., McDonald M., Rainer H., van Oosterom A., Smyth J. (1985). Mitoxantrone as first-line chemotherapy in advanced breast cancer: results of a collaborative European study.. Invest New Drugs.

[OCR_00393] Powles T. J., Coombes R. C., Smith I. E., Jones J. M., Ford H. T., Gazet J. C. (1980). Failure of chemotherapy to prolong survival in a group of patients with metastatic breast cancer.. Lancet.

[OCR_00399] Robertson J. F., Williams M. R., Todd J. H., Blamey R. W. (1989). Mitoxantrone--a useful palliative therapy in advanced breast cancer.. Am J Clin Oncol.

[OCR_00404] Robertson J. F., Williams M. R., Todd J., Nicholson R. I., Morgan D. A., Blamey R. W. (1989). Factors predicting the response of patients with advanced breast cancer to endocrine (Megace) therapy.. Eur J Cancer Clin Oncol.

[OCR_00410] Ross M. B., Buzdar A. U., Blumenschein G. R. (1982). Treatment of advanced breast cancer with megestrol acetate after therapy with tamoxifen.. Cancer.

[OCR_00417] Walker K. J., Bouzubar N., Robertson J., Ellis I. O., Elston C. W., Blamey R. W., Wilson D. W., Griffiths K., Nicholson R. I. (1988). Immunocytochemical localization of estrogen receptor in human breast tissue.. Cancer Res.

[OCR_00422] Williams M. R., Todd J. H., Nicholson R. I., Elston C. W., Blamey R. W., Griffiths K. (1986). Survival patterns in hormone treated advanced breast cancer.. Br J Surg.

